# Supplementary material for: Integrated Metabolomics and Transcriptomics Using an Optimised Dual Extraction Process to Study Human Brain Cancer Cells and Tissues
Source: Metabolites. 2021 Apr 14;11(4):240. doi: 10.3390/metabo11040240 (PMC8070957; doi:10.3390/metabo11040240)
Supplement: Supplementary file 1 [file metabolites-11-00240-s001.pdf]

Supplementary Information

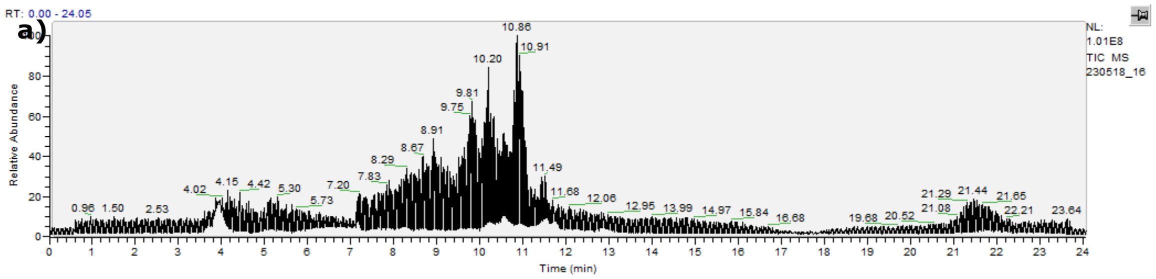

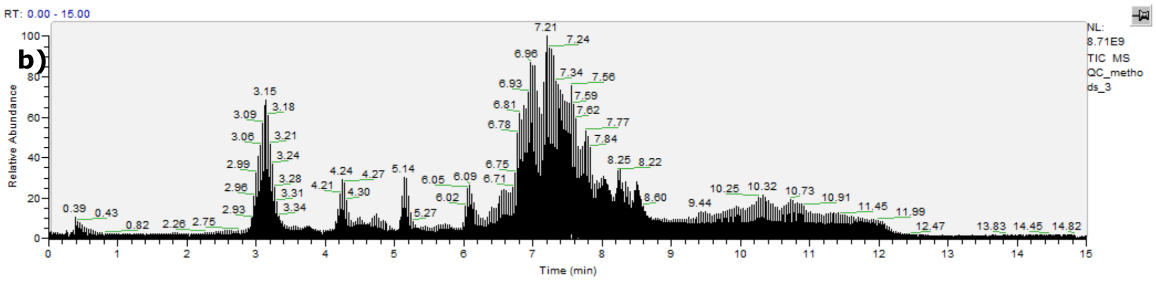

Figure S1: Total ion chromatograms of pooled QCs analyses of dual extraction methods, a) injection 2 of metabolomics QC, b) injection 3 of lipidomics QC.

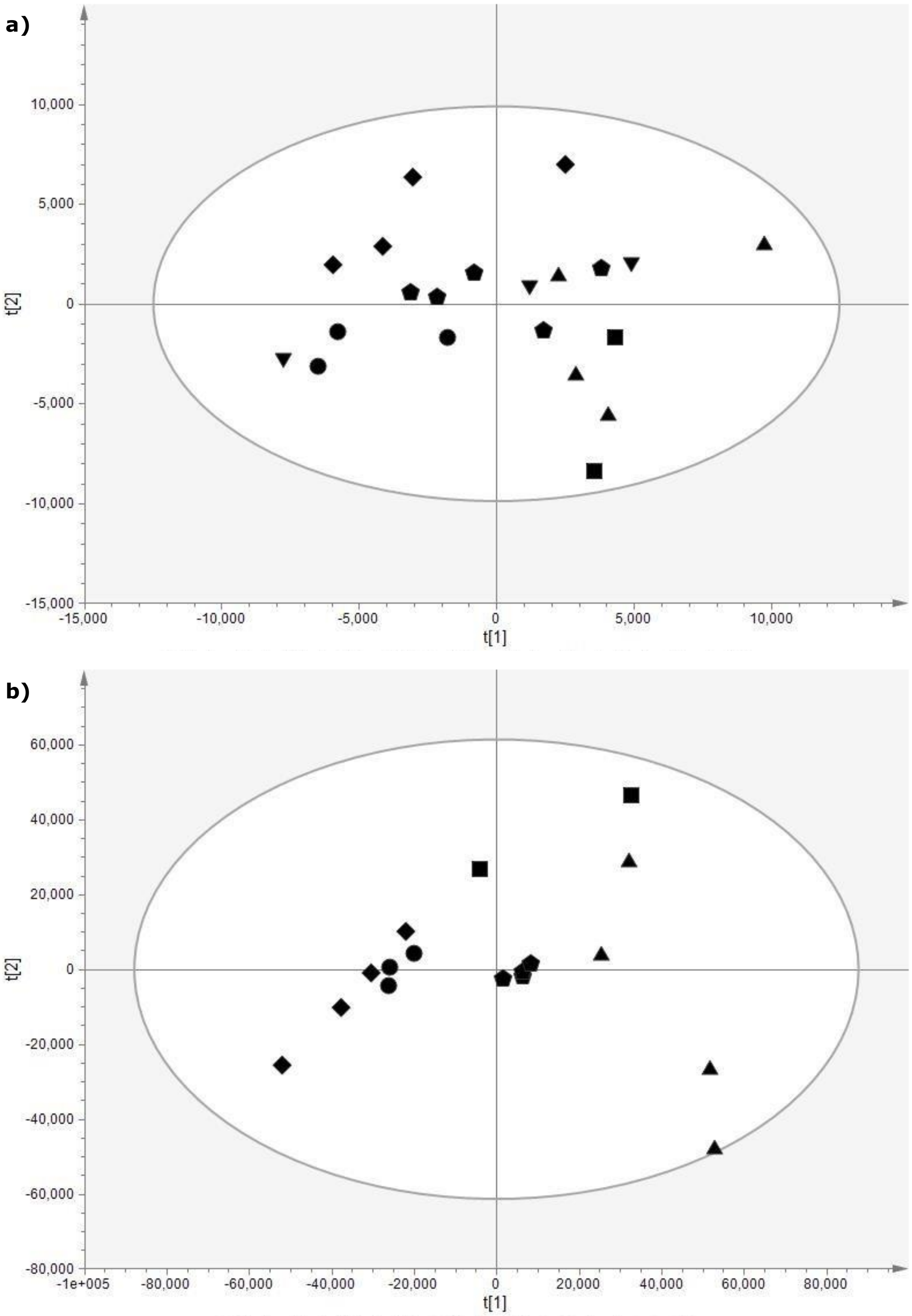

Figure S2: PCA scores plot for the metabolomics analysis (a) and lipidomics analysis (b) of the single extraction method, the four dual extraction methods and the QCs. Note that one of the four dual extraction methods did not produce an organic phase and so samples were not submitted for lipidomics analysis. a) R^2 0.518, Q^2 0.290, b) R^2 0.834, Q^2 0.559. Sequential solvent addition and shaking – ●, cryomill/mirVana – ■, cryomill-wash/Econospin – ▲, rotation/phenol-chloroform – ▼, sequential/mirVana – ◆, QC – ⬟

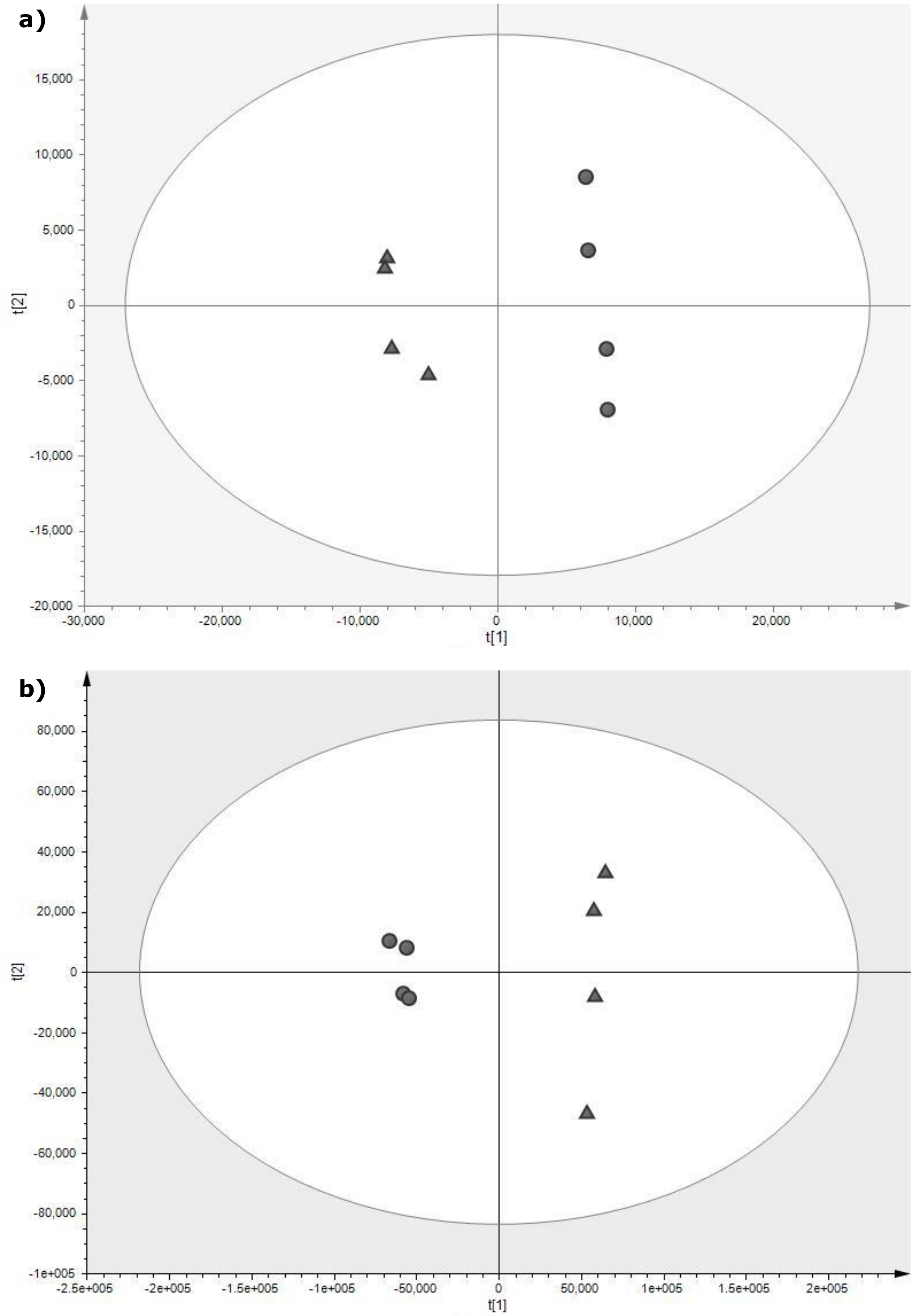

Figure S3: PCA scores plot for the metabolomics analysis (a) and lipidomics analysis (b) of the serum-starved and serum-replete BXD-1425 cells after 28 h. a) R^2 0.825, Q^2 0.660; b) R^2 0.902, Q^2 0.819. ● Control (serum-replete), ▲ treatment (serum starved).

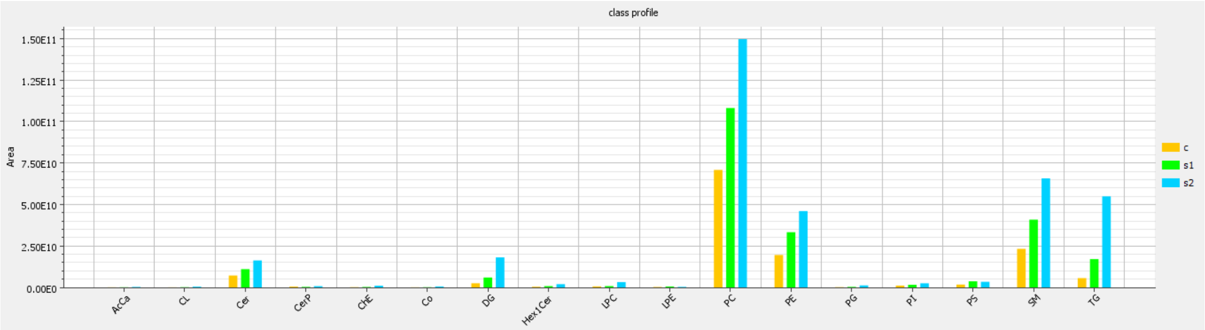

Figure S4: Lipid classes extracted from ependymoma tissue from patient 15/243 and their summed peak areas. Lipids were extracted from 10, 20 and 40 mg tissue and the six most common lipid classes were ceramides (Cer), diglycerides (DG), phosphatidylcholines (PC), phosphatidylethanolamines (PE), sphingomyelins (SM), and triglycerides.

Table S1: Differentially abundant metabolites between serum-starved (treatment) and serum-replete (control) cells. Fold changes are relative to control cells. As this was an untargeted method, peak heights, rather than concentrations, were obtained. Differences in peak height were statistically significant for all metabolites displayed. Statistical significance was defined as a combination of $p<0.05$ (in a univariate t-test with an FDR cut-off of 0.05) and VIP≥1 (variable important for projection in a multivariate OPLS-DA test). Column L is the identification level of confidence.

Metabolite	Fold-change	Accurate mass	Formula	Retention time (min)	L
Creatine	0.46	131.0695	C4H9N3O2	10.356	3
Choline phosphate	1.19	183.0661	C5H14NO4P	9.798	2
L-Proline	1.32	115.0633	C5H9NO2	9.262	2
L-Phenylalanine	0.98	165.0790	C9H11NO2	7.747	2
sn-glycero-3-Phosphocholine	1.60	257.1028	C8H20NO6P	9.655	2
L-Leucine	1.05	131.0947	C6H13NO2	8.145	2
L-Methionine	0.83	149.0510	C5H11NO2S	8.605	2
L-Valine	1.16	117.0789	C5H11NO2	8.990	2
L-Glutamate	0.98	147.0531	C5H9NO4	9.998	2
L-Glutamine	3.19	146.0690	C5H10N2O3	10.458	2
L-Threonine	1.08	119.0582	C4H9NO3	10.155	2
L-Histidine	1.05	155.0694	C6H9N3O2	10.236	2
L-1-Pyrroline-3-hydroxy-5-carboxylate	1.29	129.0427	C5H7NO3	7.749	3
L-Tyrosine	0.95	181.0740	C9H11NO3	9.678	2
(S)-Malate	0.64	134.0215	C4H6O5	11.050	2
L-Carnitine	0.32	161.1052	C7H15NO3	9.244	2

L-Serine	0.55	105.0426	C3H7NO3	10.934	2
Glutathione	0.65	307.0837	C10H17N3O6S	9.616	3
L-Alanine	0.43	89.0477	C3H7NO2	10.414	2
N-Acetyl-L-glutamate 5-semialdehyde	1.26	173.0688	C7H11NO4	4.729	3
O-Acetylcarnitine	0.14	203.1158	C9H17NO4	7.992	2
Creatinine	0.24	113.0589	C4H7N3O	7.914	2
(S)-1-Pyrroline-5-carboxylate	1.37	113.0477	C5H7NO2	10.710	3
L-Aspartate	0.83	133.0375	C4H7NO4	10.228	2
N6-Methyl-L-lysine	0.67	160.1211	C7H16N2O2	15.502	2
2-Dehydro-3-deoxy-L-rhamnonate	0.38	162.0528	C6H10O5	9.889	3
O-Propanoylcarnitine	0.26	217.1314	C10H19NO4	7.232	3
Choline	1.52	103.0997	C5H13NO	9.655	3
(R)-2-Hydroxyglutarate	0.76	148.0371	C5H8O5	10.533	2
L-Glutamate 5-semialdehyde	0.29	131.0583	C5H9NO3	10.217	3
N-Acetylputrescine	0.35	130.1106	C6H14N2O	16.284	2
N2-Acetyl-L-aminoadipate	0.38	203.0795	C8H13NO5	8.828	3
Glu-Gly	3.86	204.0747	C7H12N2O5	6.866	3
Glycine	1.27	75.0320	C2H5NO2	11.006	2
O-Acetyl-L-homoserine	0.42	161.0688	C6H11NO4	8.180	3
Ascorbate	0.30	176.0321	C6H8O6	10.780	3
N(pi)-Methyl-L-histidine	0.11	169.0851	C7H11N3O2	8.884	2

Succinate	0.37	118.0266	C4H6O4	10.544	2
Adenine	0.73	135.0545	C5H5N5	7.872	2
4-Trimethylammoniobutanoate	1.33	145.1103	C7H15NO2	9.178	2
N-Acetyl-D-glucosamine	0.37	221.0899	C8H15NO6	8.840	2
L-Citrulline	0.15	175.0957	C6H13N3O3	10.806	2
[SP hydroxy,hydroxy,methyl(10:2/2:0)] 6R-(8-hydroxydecyl)-2R-(hydroxymethyl)-piperidin-3R-ol	1.52	287.2460	C16H33NO3	6.448	3
Diacetyl	0.13	86.0368	C4H6O2	10.656	3
L-Cystathionine	0.33	222.0673	C7H14N2O4S	11.055	2
Tiglic acid	0.25	100.0524	C5H8O2	10.627	3
Iminoaspartate	0.52	131.0218	C4H5NO4	6.934	3
Asn-Asn-Asn	1.45	360.1389	C12H20N6O7	8.641	3
2-C-Methyl-D-erythritol 4-phosphate	10.32	216.0401	C5H13O7P	10.662	3
(S)-Dihydroorotate	0.06	158.0327	C5H6N2O4	8.439	3
Guanidinoacetate	0.26	117.0538	C3H7N3O2	11.238	2
N-Carbamyl-L-glutamate	0.39	190.0590	C6H10N2O5	10.889	3
4-Acetamidobutanoate	0.17	145.0739	C6H11NO3	5.056	3
L-Asparagine	3.56	132.0534	C4H8N2O3	10.585	2
N-Carbamoyl-L-aspartate	0.13	176.0433	C5H8N2O5	11.347	3
D-Galactosamine	0.06	179.0794	C6H13NO5	8.408	3
Cytidine	7.75	243.0855	C9H13N3O5	9.149	2
L-2-Aminoadipate	10.34	161.0688	C6H11NO4	10.072	2

Hydantoin-5-propionate	3.74	172.0484	C6H8N2O4	6.835	3
Cytosine	9.29	111.0433	C4H5N3O	9.139	2
3-Hydroxy-N6,N6,N6-trimethyl-L-lysine	3.18	204.1474	C9H20N2O3	13.004	3
Pyruvate	10.23	88.0160	C3H4O3	6.810	2
Leu-Lys	19.85	259.1895	C12H25N3O3	13.050	3
[PC acety] 1-acetyl-sn-glycero-3-phosphocholine	38.93	299.1134	C10H22NO7P	7.843	3

Table S2: Differentially abundant lipids between serum-starved (treatment) and serum-replete (control) cells. Fold changes are relative to control cells. As this was an untargeted method, peak heights, rather than concentrations, were obtained. Differences in peak height were statistically significant for all lipids displayed. Statistical significance was defined as a combination of $p<0.05$ (in a univariate t-test with an FDR cut-off of 0.05) and VIP≥1 (variable important for projection in a multivariate OPLS-DA test). All identifications are level 3.

Lipid	Fold-change	Accurate mass	Formula	Retention time (min)
Cer(d18:1_16:0)	0.66	537.5121	C34 H67 O3 N1	7.376
Cer(d18:1_18:0)	0.37	565.5434	C36 H71 O3 N1	7.701
Cer(d18:1_20:0)	0.37	593.5747	C38 H75 O3 N1	7.993
Cer(d16:1_23:0)	0.18	607.5903	C39 H77 O3 N1	8.127
Cer(d18:1_22:0)	0.73	621.6060	C40 H79 O3 N1	8.248
Cer(d16:1_24:1)	0.59	619.5903	C40 H77 O3 N1	8.004
Cer(d18:1_23:0)	0.35	635.6216	C41 H81 O3 N1	8.380
Cer(d18:2_23:0)	0.46	633.6060	C41 H79 O3 N1	8.151
Cer(d18:0_24:1)	1.97	649.6373	C42 H83 O3 N1	8.344
Cer(d18:1_24:0)	0.69	649.6373	C42 H83 O3 N1	8.513
Cer(d18:1_24:1)	0.75	647.6216	C42 H81 O3 N1	8.244
Cer(d18:1_24:2)	0.59	645.6060	C42 H79 O3 N1	8.043
Cer(t16:1_12:0)	1.56	469.4131	C28 H55 O4 N1	6.098
ChE(18:2)	0.14	648.5845	C45 H76 O2	10.494
DG(18:1_18:1)	0.40	620.5380	C39 H72 O5	8.017
DG(40:4e)	0.11	658.5900	C43 H78 O4	8.471
Hex1Cer(d18:1_24:0)	2.04	811.6901	C48 H93 O8 N1	8.159
LPC(16:0)	0.29	495.3325	C24 H50 O7 N1 P1	3.537
LPC(18:0)	0.26	523.3638	C26 H54 O7 N1 P1	4.453
PC(30:0)	1.45	705.5309	C38 H76 O8 N1 P1	6.824
PC(30:0e)	2.03	691.5516	C38 H78 O7 N1 P1	7.061
PC(30:1)	1.94	703.5152	C38 H74 O8 N1 P1	6.519
PC(30:2)	2.56	701.4996	C38 H72 O8 N1 P1	6.365
PC(31:0)	1.84	719.5465	C39 H78 O8 N1 P1	7.011
PC(31:1)	2.04	717.5309	C39 H76 O8 N1 P1	6.716
PC(32:0)	1.44	733.5622	C40 H80 O8 N1 P1	7.170
PC(32:0e)	1.79	719.5829	C40 H82 O7 N1 P1	7.399
PC(32:1)	1.52	731.5465	C40 H78 O8 N1 P1	6.897
PC(32:1e)	1.67	717.5672	C40 H80 O7 N1 P1	7.134
PC(14:0e_18:1)	1.91	717.5672	C40 H80 O7 N1 P1	7.367
PC(32:3)	1.83	727.5152	C40 H74 O8 N1 P1	6.493
PC(34:0e)	1.46	747.6142	C42 H86 O7 N1 P1	7.710
PC(16:0_18:1)	1.54	759.5778	C42 H82 O8 N1 P1	7.225
PC(34:1e)	1.38	745.5985	C42 H84 O7 N1 P1	7.443

PC(16:0_18:2)	1.30	757.5622	C42 H80 O8 N1 P1	6.982
PC(34:2)	1.39	757.5622	C42 H80 O8 N1 P1	7.261
PC(16:1e_18:1)	1.62	743.5829	C42 H82 O7 N1 P1	7.410
PC(35:1)	1.44	773.5935	C43 H84 O8 N1 P1	7.889
PC(36:0)	0.44	789.6248	C44 H88 O8 N1 P1	7.800
PC(18:0_18:1)	1.36	787.6091	C44 H86 O8 N1 P1	7.552
PC(36:1)	1.24	787.6091	C44 H86 O8 N1 P1	7.936
PC(18:1_18:1)	1.32	785.5935	C44 H84 O8 N1 P1	7.312
PC(36:2)	1.26	785.5935	C44 H84 O8 N1 P1	7.609
PC(36:2e)	1.59	771.6142	C44 H86 O7 N1 P1	7.505
PC(20:4e_16:0)	1.28	767.5829	C44 H82 O7 N1 P1	7.168
PC(15:0_22:4)	1.36	795.5778	C45 H82 O8 N1 P1	7.613
PC(38:3)	1.33	811.6091	C46 H86 O8 N1 P1	7.396
PC(20:4e_18:0)	1.59	795.6142	C46 H86 O7 N1 P1	7.499
PE(18:2e_16:0)	2.14	701.5359	C39 H76 O7 N1 P1	7.507
PE(18:0_18:1)	1.24	745.5622	C41 H80 O8 N1 P1	7.640
PE(18:0p_18:1)	2.63	729.5672	C41 H80 O7 N1 P1	7.794
PE(18:1_18:1)	1.45	743.5465	C41 H78 O8 N1 P1	7.416
PE(16:0p_20:4)	2.54	723.5203	C41 H74 O7 N1 P1	7.247
PE(16:0p_22:3)	1.76	753.5672	C43 H80 O7 N1 P1	7.681
PE(18:0_20:4)	1.53	767.5465	C43 H78 O8 N1 P1	7.395
PE(16:0p_22:4)	1.50	751.5516	C43 H78 O7 N1 P1	7.478
PE(18:1_20:4)	1.43	765.5309	C43 H76 O8 N1 P1	7.107
PE(16:0p_22:5)	2.23	749.5359	C43 H76 O7 N1 P1	7.293
PE(18:0p_22:4)	1.63	779.5829	C45 H82 O7 N1 P1	7.760
PE(18:1p_22:4)	1.46	777.5672	C45 H80 O7 N1 P1	7.552
PI(18:0_20:4)	2.41	886.5571	C47 H83 O13 N0 P1	6.940
SM(d32:0)	0.49	676.5519	C37 H77 O6 N2 P1	6.549
SM(d34:0)	0.63	704.5832	C39 H81 O6 N2 P1	6.946
SM(d41:1)	0.43	800.6771	C46 H93 O6 N2 P1	7.953
SM(d42:2)	0.68	812.6771	C47 H93 O6 N2 P1	7.811
SM(d42:3)	0.72	810.6615	C47 H91 O6 N2 P1	7.598
TG(16:0_14:0_16:1)	0.35	776.6894	C49 H92 O6	9.711
TG(16:0_14:0_18:1)	0.51	804.7207	C51 H96 O6	10.167
TG(16:1_14:0_18:1)	0.24	802.7050	C51 H94 O6	9.793
TG(16:1_14:0_18:2)	0.11	800.6894	C51 H92 O6	9.466
TG(18:0_16:0_16:0)	0.41	834.7676	C53 H102 O6	11.288
TG(16:0_16:0_18:1)	0.70	832.7520	C53 H100 O6	10.703
TG(16:0_16:1_18:1)	0.44	830.7363	C53 H98 O6	10.259
TG(18:1_14:0_18:2)	0.17	828.7207	C53 H96 O6	9.857
TG(14:0_18:2_18:2)	0.14	826.7050	C53 H94 O6	9.566
TG(18:0_16:0_18:1)	0.51	860.7833	C55 H104 O6	11.346
TG(16:0_18:1_18:1)	0.51	858.7676	C55 H102 O6	10.775
TG(16:0e_18:1_18:1)	0.24	844.7884	C55 H104 O5	11.761

TG(16:0_18:1_18:2)	0.28	856.7520	C55 H100 O6	10.331
TG(16:1_18:1_18:2)	0.10	854.7363	C55 H98 O6	9.954
TG(16:1_18:2_18:2)	0.14	852.7207	C55 H96 O6	9.757
TG(16:0_18:1_20:1)	0.46	886.7989	C57 H106 O6	11.421
TG(18:1_18:1_18:1)	0.33	884.7833	C57 H104 O6	10.870
TG(18:1_18:1_18:2)	0.27	882.7676	C57 H102 O6	10.398
TG(18:1e_16:0_20:3)	0.10	868.7884	C57 H104 O5	11.517
TG(18:1_18:2_18:2)	0.19	880.7520	C57 H100 O6	10.213
TG(18:2_18:2_18:2)	0.13	878.7363	C57 H98 O6	9.851
TG(16:0_20:2_20:2)	0.34	910.7989	C59 H106 O6	11.077
TG(18:1_18:1_20:3)	0.20	908.7833	C59 H104 O6	10.644
TG(18:1e_16:0_22:4)	0.10	894.8040	C59 H106 O5	11.582
TG(18:1_18:2_20:3)	0.17	906.7676	C59 H102 O6	10.260
TG(18:1_18:2_20:4)	0.10	904.7520	C59 H100 O6	9.917
TG(18:0_18:1_22:4)	0.22	936.8146	C61 H108 O6	11.245
TG(18:1_18:1_22:4)	0.16	934.7989	C61 H106 O6	10.717
TG(18:1e_18:1_22:4)	0.10	920.8197	C61 H108 O5	11.774
TG(18:1_20:3_20:3)	0.10	932.7833	C61 H104 O6	10.331
TG(20:3_18:2_20:3)	0.08	930.7676	C61 H102 O6	10.108

Table S3: Gene/metabolite nodes and edge interactions of metabolic pathways upon serum-starvation of BXD-1425 ependymoma cells. Metabolomic/transcriptomic data integration identified interactions occurring within 37 metabolic pathways.

Metabolic Pathways	Gene Nodes	Metabolite Nodes	Edge Interactions	Significant Genes	Significant Metabolites	Significant Interactions
Aminosugars metabolism	5	6	13	YES	YES	YES
Androgen and estrogen biosynthesis and metabolism	26	8	208	YES	NO	NO
Arachidonic acid metabolism	26	20	231	YES	NO	NO
Bile acid biosynthesis	2	5	8	NO	YES	NO
Biopterin metabolism	1	4	4	NO	YES	NO
Butanoate metabolism	2	2	4	NO	YES	NO
C21-steroid hormone biosynthesis and metabolism	26	7	182	YES	NO	NO
Galactose metabolism	4	3	12	NO	YES	NO
Glycerophospholipid metabolism	17	13	51	NO	YES	NO
Glycine, serine, alanine and threonine metabolism	42	47	120	NO	YES	NO
Glycolysis and Gluconeogenesis	13	19	56	NO	YES	NO
Glycosphingolipid biosynthesis - ganglioseries	5	4	20	YES	NO	NO
Glycosphingolipid metabolism	2	3	6	NO	YES	NO
Histidine metabolism	7	11	19	NO	YES	NO
Leukotriene metabolism	29	11	171	YES	YES	NO
Linoleate metabolism	29	4	84	YES	NO	NO
Lysine metabolism	30	14	97	YES	YES	NO
Methionine and cysteine metabolism	13	17	37	NO	YES	NO
Omega-3 fatty acid metabolism	3	4	12	YES	NO	NO
Omega-6 fatty acid metabolism	3	4	12	YES	NO	NO

Pentose phosphate pathway	2	2	4	YES	NO	NO
Phytanic acid peroxisomal oxidation	1	3	3	NO	YES	NO
Porphyrin metabolism	2	4	8	NO	YES	NO
Prostaglandin formation from arachidonate	2	2	4	YES	NO	NO
Proteoglycan biosynthesis	14	4	46	YES	NO	NO
Purine metabolism	138	19	451	YES	YES	NO
Pyrimidine metabolism	39	14	104	NO	YES	NO
Selenoamino acid metabolism	5	2	10	YES	NO	NO
TCA cycle	10	5	20	NO	YES	NO
Tryptophan metabolism	31	5	72	YES	NO	NO
Tyrosine metabolism	63	21	185	YES	YES	NO
Urea cycle and metabolism of arginine, proline, glutamate, aspartate and asparagine	100	66	322	YES	YES	YES
Valine, leucine and isoleucine degradation	6	8	20	NO	YES	NO
Vitamin A (retinol) metabolism	6	2	12	YES	NO	NO
Vitamin B3 (nicotinate and nicotinamide) metabolism	1	3	3	NO	YES	NO
Vitamin B9 (folate) metabolism	5	11	20	YES	YES	NO
Xenobiotics metabolism	26	44	114	YES	NO	NO

Table S4: Metabolites present in the brain tumour of patient 15/243. Fold changes are relative to the 10 mg portion of tumour. As this was an untargeted method, peak heights, rather than concentrations, were obtained. Column L is the identification level of confidence.

Metabolite	Fold-change 20 mg	Fold-change 40 mg	Accurate mass	Formula	Retention time (min)	L
Creatinine	1.75	3.05	113.059	C4H7N3O	8.02	2
O-Acetylcarnitine	1.92	2.01	203.116	C9H17NO4	8.06	2
L-Leucine	1.76	3.2	131.095	C6H13NO2	8.35	2
Betaine	1.34	1.71	117.079	C5H11NO2	8.58	2
L-Methionine	1.43	2.46	149.051	C5H11NO2S	8.73	2
L-Tryptophan	1.9	3.73	204.09	C11H12N2O2	9.15	2
4-Trimethylammoniobutanoate	1.43	2.31	145.11	C7H15NO2	9.27	2
L-Carnitine	1.26	2.35	161.105	C7H15NO3	9.33	2
L-Proline	1.49	2.52	115.063	C5H9NO2	9.45	2
L-Tyrosine	1.62	2.36	181.074	C9H11NO3	9.78	2
L-Glutamate	1.5	2.2	147.053	C5H9NO4	10.09	2
L-2-Aminoadipate	1.34	2.1	161.069	C6H11NO4	10.17	2
L-Threonine	1.58	2.3	119.058	C4H9NO3	10.19	2
L-Aspartate	1.81	3.08	133.037	C4H7NO4	10.38	2
L-Alanine	1.32	1.52	89.048	C3H7NO2	10.44	2
L-Glutamine	1.31	1.22	146.069	C5H10N2O3	10.5	2
L-Histidine	1.71	2.95	155.069	C6H9N3O2	10.56	2
L-Asparagine	1.57	1.54	132.053	C4H8N2O3	10.64	2
L-Serine	2.18	3.83	105.043	C3H7NO3	11.02	2
Glycine	1.57	2.69	75.032	C2H5NO2	11.07	2
L-Cystathionine	3.37	6.24	222.067	C7H14N2O4S	11.15	2
Guanidinoacetate	1.3	2.39	117.054	C3H7N3O2	11.28	2
N6-Methyl-L-lysine	1.63	4.46	160.121	C7H16N2O2	15.85	2
L-Arginine	1.96	3.48	174.112	C6H14N4O2	17.67	2
D-Glycerate	1.29	1.79	106.027	C3H6O4	8.99	2
N-Acetylneuraminate	1.1	1.56	309.106	C11H19NO9	9.31	2
D-Glucose 6-phosphate	1.89	3.91	260.03	C6H13O9P	10.81	2
myo-Inositol	2.31	3.85	180.063	C6H12O6	11.77	2
sn-glycero-3-Phosphocholine	1.81	1.89	257.103	C8H20NO6P	9.7	2
Taurine	1.53	2.84	125.015	C2H7NO3S	10.92	2

O-Butanoylcarnitine	1.18	2.47	231.147	C11H21NO4	6.9	2
Nicotinamide	1.57	3.15	122.048	C6H6N2O	6.58	2
Uridine	1.34	1.79	244.069	C9H12N2O6	8.23	2
Orotate	2.59	3.48	156.017	C5H4N2O4	8.37	2
Hypoxanthine	1.56	2.07	136.038	C5H4N4O	8.53	2
Inosine	1.49	2.56	268.081	C10H12N4O5	8.68	2
Xanthine	1.3	1.13	152.033	C5H4N4O2	9.09	2
Xanthosine	1.03	2.62	284.075	C10H12N4O6	9.22	2
Urate	1.32	1.57	168.028	C5H4N4O3	9.56	2
L-Phenylalanine	1.53	3.13	165.079	C9H11NO2	7.88	3
L-1-Pyrroline-3-hydroxy-5-carboxylate	1.23	2.09	129.043	C5H7NO3	7.93	3
[FA hydroxy,oxo(7:0/2:0)] 4-hydroxy-2-oxo-Heptanedioic acid	1.36	2.5	190.048	C7H10O6	8.64	3
L-Pipecolate	1.71	2.51	129.079	C6H11NO2	9.15	3
L-Methionine S-oxide	1.77	1.8	165.046	C5H11NO3S	9.32	3
N2-Acetyl-L-aminoadipate	1.47	3.33	203.079	C8H13NO5	9.53	3
Glutathione	1.45	1.5	307.084	C10H17N3O6S	9.72	3
O-Succinyl-L-homoserine	1.67	2.74	219.074	C8H13NO6	9.86	3
N-Acetyl-L-glutamate	1.35	2.7	189.064	C7H11NO5	9.92	3
N-Acetyl-L-aspartate	0.8	2.36	175.048	C6H9NO5	10.25	3
Creatine	1.4	1.78	131.069	C4H9N3O2	10.43	3
Hypotaurine	1.56	2.9	109.02	C2H7NO2S	10.65	3
4-Guanidinobutanoate	1.44	2.65	145.085	C5H11N3O2	10.66	3
Choline	1.42	2.38	103.1	C5H13NO	16.9	3
D-Galactosamine	1.45	2.05	179.079	C6H13NO5	8.44	3
2,5-Dioxopentanoate	1.21	2.61	130.027	C5H6O4	8.87	3
[FA trihydroxy(4:0)] 2,3,4-trihydroxy-butanoic acid	1.66	1.93	136.037	C4H8O5	9.38	3
Citrate	1.33	2.26	192.027	C6H8O7	9.39	3
Orthophosphate	1.81	3.21	97.977	H3O4P	10.8	3
Triethanolamine	1.83	2.05	149.105	C6H15NO3	7.73	3
sn-glycero-3-Phosphoethanolamine	1.56	1.29	215.056	C5H14NO6P	10.5	3

[FA (12:4/2:0)] 2E,4E,8E,10E-Dodecatetraenedioic acid	0.88	1.48	222.089	C12H14O4	4.16	3
[FA (6:0)] O-hexanoyl-R-carnitine	1.15	2.21	259.179	C13H25NO4	4.93	3
O-Propanoylcarnitine	2.23	3.31	217.131	C10H19NO4	7.44	3
Orotate(Fragment)	1.24	2.02	112.027	C4H4N2O2	8.25	3

Table S5: Number of lipid groups in each class and total number of lipid groups extracted from 10, 20 and 40 mg ependymoma tissue from patient 15/243. A lipid group is a group of lipids who whave the same fatty acid number, e.g. all phosphatidylcholines which have two fatty acids totalling (36:2) will be one lipid group.

Class	Class name	10 mg sample	20 mg sample	40 mg sample
CL	cardiolipin	1	3	11
LPC	lysophosphatidylcholine	3	3	5
PC	phosphatidylcholine	59	67	78
LPE	lysophosphatidylethanolamine	3	8	4
PE	phosphatidylethanolamine	32	46	43
PG	phosphatidylglycerol	0	6	15
PI	phosphatidylinositol	8	8	10
PS	phosphatidylserine	4	7	7
Cer	ceramides	18	19	20
CerP	ceramides phosphate	0	0	1
Hex1Cer	simple Glc series	5	6	6
SM	sphingomyelin	24	29	32
ChE	cholesterol ester	1	2	6
DG	diglyceride	13	15	18
TG	triglyceride	26	36	62
AcCa	acyl carnitine	1	1	5
AEA	N-Acylethanolamine	1	1	1
Co	coenzyme	1	1	1
Total number:		200	258	325

Table S6: Total peak area per class of lipids, extracted from 10, 20 and 40 mg ependymoma tissue from patient 15/243. Table also shows whether the class area at 40 mg deviates from linearity (i.e. saturation is observed).

Class	Class name	Class area 10 mg sample	Class area 20 mg sample	Class area 40 mg sample	Detector or solution saturated at 40 mg?
AcCa	acyl carnitine	6.77E+07	1.03E+08	3.09E+08	no
CL	cardiolipin	5.72E+07	1.46E+08	4.81E+08	no
Cer	ceramides	7.18E+09	1.10E+10	1.63E+10	yes
CerP	ceramides phosphate	5.18E+08	3.44E+08	7.59E+08	-
ChE	cholesterol ester	1.48E+08	2.93E+08	1.06E+09	no
Co	coenzyme	6.73E+07	1.31E+08	5.90E+08	no
DG	diglyceride	2.51E+09	5.98E+09	1.81E+10	no
Hex1Cer	simple Glc series	4.24E+08	7.89E+08	1.96E+09	no
LPC	lysophosphatidylcholine	6.16E+08	8.00E+08	3.23E+09	no
LPE	lysophosphatidylethanolamine	2.64E+08	5.79E+08	3.39E+08	yes
PC	phosphatidylcholine	7.08E+10	1.08E+11	1.50E+11	yes
PE	phosphatidylethanolamine	1.95E+10	3.33E+10	4.60E+10	yes
PG	phosphatidylglycerol	1.54E+08	3.48E+08	1.23E+09	no
PI	phosphatidylinositol	1.13E+09	1.59E+09	2.49E+09	no

PS	phosphatidylserine	1.72E+09	3.70E+09	3.40E+09	yes
SM	sphingomyelin	2.32E+10	4.09E+10	6.57E+10	yes
TG	triglyceride	5.61E+09	1.71E+10	5.49E+10	no

Supplementary Methods – Expanded methods

Cryomill/mirVana Method: Autoclaved-sterilized milling balls (5x2 mm+2x5 mm) were cooled inside tubes in liquid nitrogen. They were added to the frozen cell pellet which was cryo-milled for 2 minutes at 25 Hz in a Mixer Mill 301 (Retsch) by pre-cooling the adapter rack in liquid nitrogen. To keep the resulting powder frozen, the tube was dipped immediately into liquid nitrogen after milling. Cold (4 °C) methanol/water (1:1, v/v, 300 µl) and cold chloroform (300 µl) were added consecutively. The solution was vortexed until the powdered sample had dispersed into the solvent mixture. The sample was cryo-milled in a Mixer Mill 301 (Retsch) for 2 minutes at 20 Hz. The Eppendorf tube cap was wrapped with Parafilm before cryomilling to create a tight seal to prevent solvent leakage. The sample was centrifuged at 14 000 rcf and 4 °C for 5 minutes to separate the phases. Polar and nonpolar phases were transferred into new Eppendorf tubes and stored at -80 °C until the solvents were evaporated on the Jouan Centrifugal Evaporator at room temperature and then the residues stored at -80 °C**.** The interphase with the milling beads was placed on ice and the RNA was extracted using the mirVana™ miRNA Isolation Kit (Ambion) as outlined above**.**

Cryomill-wash/Econospin Method: To the frozen cell pellet, 500 µl of methanol, 200 µl of chloroform and 100 µl of water were added, all at 4 °C. Cooled autoclaved-sterilized milling balls (5x2 mm+2x5 mm) were added to the mix. The sample was cryo-milled in a Mixer Mill 301 (Retsch) for 30 seconds at 25 Hz. The sample was centrifuged at 20 000 rcf for 6 minutes at 4 °C. To a 2 ml Eppendorf tube containing 800 µl of phase separation mix (chloroform:water 1:1), the supernatant was added and vortexed to mix. The pellet, containing proteins and nucleic acids, was immediately washed with 1 ml of 0.75% (v/v) β-mercaptoethanol in 100% methanol.

The tube with metabolites was centrifuged at 10 000 rcf for 5 minutes at room temperature. The upper polar phase and lower non-polar phase were transferred into separate Eppendorf tubes, to which were added 300 µl of phase separation mix. This enabled sparingly soluble molecules to be washed out. These two tubes were centrifuged and the polar phase from one tube and the nonpolar phase from the second tube were each transferred into fresh Eppendorf tubes and stored at -80 °C until the solvents were evaporated on the Jouan Centrifugal Evaporator at room temperature. Residues were stored at -80 °C**.**

The tube containing the pellet was centrifuged at 20 000 rcf for 6 minutes at 4 °C and the supernatant was removed gently by pipette and discarded. The pellet was washed a second time with 1 ml of 0.75% (v/v) β-mercaptoethanol in 100% methanol, centrifuged, and the supernatant discarded. The pellet was air dried in the fume cupboard, then dissolved in 400 µl of pellet solubilisation buffer (7 M guanidine HCl, 2% (v/v) Tween-20, 4% (v/v)

Triton-x100, 50 mM Tris, pH 7.5, 1% (v/v) β-mercaptoethanol). The tube was shaken (300 rpm) at 37°C until the pellet had thoroughly dissolved (30 minutes). The sample was transferred into a new tube, leaving the milling balls behind, and centrifuged at 14 000 rcf for 3 minutes to sediment anything insoluble. The supernatant was pipetted onto a new silica column (Econospin, 1940-250, Epoch Life Science) to bind DNA. After leaving to rest for 1 minute, the column was centrifuged at 10 000 rcf for 1 minute. Acetonitrile (300 µl) was instantly mixed into the flowthrough to aid total RNA extraction, which was then quickly transferred to a new silica column. After leaving to rest for 1 minute, the column was centrifuged at 10 000 rcf for 1 minute and the flowthrough discarded. Both columns were washed with 750 µl of wash buffer 1 (2 mM Tris pH 7.5, 20 mM NaCl, 0.1 mM EDTA, 90% ethanol), then centrifuged at 12 000 rcf for 2 minutes, after which the flowthrough was discarded. Columns were then washed with 750 µl of wash buffer 2 (2 mM Tris pH 7.5, 20 mM NaCl, 70% ethanol), then centrifuged at 12 000 rcf for 2 minutes and the flowthrough discarded. An additional centrifugation step for 1 minute at 14 000 rcf was taken to fully dry the columns. The nucleic acids were eluted from the column in 50 µl of DNA elution buffer (for DNA samples, 10 mM Tris, pH 8.0, 1 mM EDTA) or nuclease-free water (for RNA samples).

Rotation/phenol-chloroform Method: A frozen cell pellet was re-suspended in PBS (50 µl), then further re-suspended in a methanol/chloroform/water solution (9:1:10, v/v, 1 ml). The sample tube was rotated for 30 min in the cold room (4 °C) by placing it vertically inside a larger tube on a roller. The sample was centrifuged at 500 rcf at 4 °C for 10 minutes. The metabolite-containing supernatant was transferred into a new Eppendorf tube and stored at -80 °C until the solvent was evaporated on the Jouan Centrifugal Evaporator at room temperature and residues stored at -80 °C. The RNA-containing pellet was re-suspended in 1x PBS (100 µl). Water saturated phenol (1 ml) was added to separate the RNA, DNA and proteins. The sample tube was rotated for 5 minutes in the cold room (4 °C). Chloroform (200 µl) was added to separate the phases and the tube was placed at room temperature for 5 minutes. The sample was centrifuged at 12 000 rcf for 15 minutes at 4 °C. The upper RNA-containing phase was transferred to a new tube, whereupon it was mixed with ice-cold isopropanol (500 µl) and left at room temperature for 10 minutes. Following centrifugation for 20 minutes at 12 000 rcf at 4 °C, the supernatant was removed gently by pipette. The RNA pellet was washed in 75% (v/v) ethanol (1 ml) and the solution was centrifuged for a further 20 minutes at 12 000 rcf at 4 °C. The supernatant was again removed gently by pipette. The pellet was air-dried for 10-15 minutes at RT and finally re-suspended in 15 µl of nuclease-free water (60 °C).
